# Architecture for Multi-Technology Real-Time Location Systems

**DOI:** 10.3390/s130202220

**Published:** 2013-02-07

**Authors:** Javier Rodas, Valentín Barral, Carlos J. Escudero

**Affiliations:** Department of Electronics and Systems, University of A Coruna, Campus de Elviña s/n, 15071 A Coruna, Spain; E-Mails: valentin.barral@udc.es (V.B.); escudero@udc.es (C.J.E.)

**Keywords:** architecture, RTLS, data fusion, multi-technology, fingerprinting, WSN, WiFi, ZigBee, UWB, accelerometer

## Abstract

The rising popularity of location-based services has prompted considerable research in the field of indoor location systems. Since there is no single technology to support these systems, it is necessary to consider the fusion of the information coming from heterogeneous sensors. This paper presents a software architecture designed for a hybrid location system where we can merge information from multiple sensor technologies. The architecture was designed to be used by different kinds of actors independently and with mutual transparency: hardware administrators, algorithm developers and user applications. The paper presents the architecture design, work-flow, case study examples and some results to show how different technologies can be exploited to obtain a good estimation of a target position.

## 1. Introduction

The problem of real time location systems (RTLS) in indoor scenarios is receiving a great deal of attention because of the many Location-Based Services (LBSs) that can be implemented with them, such as route management [1], guidance [2], points of interest [3,4], augmented reality [5], healthcare [6] and others. To provide such services we need a positioning system, typically based on radio range communications. The Global Positioning System (GPS) is frequently used outdoors due to its worldwide coverage. Unfortunately, GPS radio waves are unable to penetrate building structures, leaving indoor areas uncovered [7]. Moreover, GPS has a poor accuracy, typically in the order of 10 meters [8,9]. Thus, other technologies such as Wireless Sensor Networks (WSN) are needed to implement Indoor Positioning Systems (IPSs) and enable LBSs in indoor environments.

Nowadays, there is a continuous emergence of many kinds of portable devices, such as mobile phones, smart-phones, PDAs, tablets and laptops, that are equipped with several radio interfaces such as WiFi, Bluetooth, GPS, NFC, and even inertial sensors, such as accelerometer and digital compass. Luckily, we can take advantage of these features for indoor positioning, and use them to directly estimate the position of persons, animals, vehicles, *etc.*

While outdoor positioning systems typically operate without many problems using satellite systems, indoor positioning is a non-trivial task, as signals that propagate through the indoor environment are scattered, reflected and affected by multi-path fading. Moreover, signals are affected by transient effects such as human bodies absorbing signals [10] or the humidity level. For instance, narrowband technologies working on the 2.4 GHz ISM band, such as WiFi, ZigBee and Bluetooth, are affected in a similar manner by environmental phenomena. They use Receive Signal Strength (RSS) noisy information for ranging and they have similar accuracy. According to the RADAR project [11] a median accuracy of fingerprint technique in a noisy WiFi channel can be of 2.94 m. Subsequent research efforts have tried to improve positioning accuracy by means of several kinds of location and classification algorithms, such as Support Vector Machines [12], Neural Networks [13,14] and Particle Filters [15–17]. Motion models have also been used to better model signal fluctuations in non-static scenarios [15–19]. However, the accuracy that can be obtained by using an indoor positioning system cannot be only attributed exclusively to the chosen location algorithm; it is also affected by factors such as the number of sensors, their placement, their sensitivity, the orientation of their antennas or the number of samples used, etc. [18,20]. Elnahrawy et al. [21] made an extensive study comparing a wide range of location algorithms, and concluded that 10 feet (about 3 m) supposes a feasible lower bound for these indoor systems based on RSS measurements, due to the inherent limitations of distinguishing RSS values at short distances.

Thus, for an LBS demanding greater accuracy, e.g., below one meter, other non-RSS based technologies are needed. Ultrasound and Ultra Wide Band (UWB) technologies appear as feasible options as they use Time-of-Arrival (ToA) and Time-Difference-of-Arrival (TDoA) information, much more precise than RSS, and more robust to multi-path fading and to external interferences. However, the availability of these technologies and their price compared, for instance, with WiFi and Bluetooth, sometimes force us to deploy multiple WSNs to cover a large scenario. UWB platforms require the deployment of a much more expensive infrastructure especially designed for precise time synchronization between the readers of the network, but on the other hand, it typically uses inexpensive UWB transmitting tags. Another weak point of UWB is that there is still no portable device on the market (*i.e.*, a smartphone) that integrates any UWB part. Luckily, the size and weight of the UWB tags are extremely small so they can be easily attached to any other portable device, being able to obtain a much higher accuracy.

To obtain a ubiquitous and reliable RTLS supporting indoor and outdoor scenarios, we require a hybrid location system supporting multiple technologies simultaneously. In this paper, we propose an approach to this problem by introducing a client-server architecture for developing a generic and easy-to-use hybrid real-time location system. The proposed system is able to obtain measurements from several WSNs (RF nodes) and several generic sensors (*i.e.*, inertial sensors, motion detector, video cameras, *etc.*), obtaining position estimations by means of several location algorithms running simultaneously in real-time. It also provides a flexible data fusion module to furnish combined positioning information to several concurrent client applications. This fusion module can automatically merge positioning information from an arbitrary number of nodes (several WSN technologies) and sensors registered in the system. Moreover, communication between the clients and the server is made by using a well defined interface.

This paper is organised as follows. Section 2 introduces an overview of the state of the art and the proposed architecture features. Section 3 presents a logic view of the elements that make up the architecture and how they are connected. Section 4 shows a runtime view of the architecture, presenting the common workflow of tasks like the registration of WSN hardware, mobile nodes, sensors, *etc.*, and the insert and query of measurements and position estimation information. Section 5 shows two implementation example case studies; one based on two ZigBee and UWB networks, and an inertial sensor (accelerometer) to illustrate how to deploy a representative set of WSN technologies, nodes and sensors in a scenario; and another real scenario showing how to implement a real fingerprinting solution based on WiFi technology. Section 6 shows some validation results using a simulated environment where we deploy two ZigBee and UWB WSNs, and we track the position of a multi-technology target, comparing several data fusion methods. Finally, Section 7 shows some conclusions and future lines of work.

## 2. State of the Art and Proposed Architecture Features

Typically location-aware applications build the entire system (including sensing, representation, and application logic) as a monolithic structure. These monolithic systems are a fast-developing solution when considering a particular application and type of sensors, but they lack flexibility and scalability. Therefore, these solutions are difficult to generalize and retarget to new applications, increasing the costs of a further development. Authors in [22] were one of the first groups who introduced the idea of a layered architecture for LBS. The seven layers try to provide a stack for any kind of location-based service or application.

In a more general way [23] described the LBS by means of a three layer model: positioning, middleware and application layer. The application layer determines the data usage for specific location applications, based on the data extracted from the middleware. The middleware provides the interfaces for the application layer, hiding technical details from the positioning layer. Finally, the positioning layer deals with the deployment, configuration and calibration of wireless location infrastructure; gathers raw data (radio signal strength, time of arrival, angle of arrival…); and calculates location data using location algorithms such as proximity, ranging, triangulation, or signal strength maps. RTLS platforms, like the one introduced in the paper, cover the positioning and middleware layers of an LBS.

Once it is clear that an architecture model for RTLS was necessary, the International Organization for Standarization (ISO) defined two standards: ISO24730-1:2006 [24] and ISO19762-5:2008 [25]. The first defines an API (Application Programming Interface) describing an RTLS service and its access methods, to enable client applications to interface with the RTLS, whilst the second provides a harmonized vocabulary with terms and definitions unique to locating systems in the area of automatic identification and data capture techniques. This glossary of terms enables communication between non-specialist users and specialists in locating systems through a common understanding of basic and advanced concepts.

We can find several examples of location systems with standalone solutions. As we can see, these systems provide particular solutions for an RTLS. Representative examples of them are:

- Authors in [26–28] take advantage of data coming from only one technology (one type of sensor) for obtaining a monolithic solution.
- Authors in [4,6] use several technologies, but they were defined for specific applications, in particular for hospitals and museums.
- Other examples of proprietary RTLS with a business model are shown in [29–32], where the owner companies provide closed solutions for their customers, relying on their own hardware and presenting large numbers of constraints in the location data provided.

The best example of an RTLS platform that works seamlessly with several technologies can be found in the LocON project [33,34]. This platform provides an interface for the end user that interacts with an application layer independent of and transparent to the supported technologies [35,36]. The defined API in LocON is oriented to application support, but it lacks interfaces for users of the platform at other levels of abstraction, *i.e.*, hardware providers or algorithm developers.

**Figure 1.**
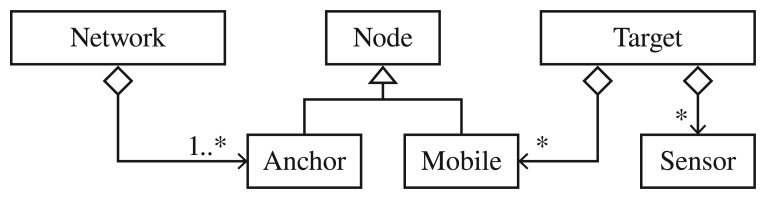
UML class diagram of the system nodes, sensors, networks, *etc.*

Taking into account this state of the art, we have considered the following requirements for the proposed architecture that improves the deficiencies of the previous solutions:

- **Technology Independent:** it must support inputs from any kind of measurement (RSS, TOA, TDOA, Angle-of-Arrival (AOA) and raw distance), generic data from any sensor (*i.e.*, inertial sensor such as accelerometer, gyroscope, digital compass, motion detector, image frames from a digital camera, *etc.*).
  Table 1 shows the standardized type of measurements supported by the proposed architecture, to consider any kind of sensor data. *The custom defined data* of a generic sensor can be modelled as an aggregation of parameters of any basic data type: float, integer, strings, boolean or binary data (encoded as string using a method such as Base64, UUEncoding, *etc.*). Or it can be modelled as an arbitrary long array-list of such kind of parameters. Section 5 shows two example case studies and it deeply explains the capabilities of the platform with generic sensors.
- **Multi-Technology:** a target to be located could be comprised of several technologies at the same time. In this way, the requirement of supporting several technologies can be used to merge heterogeneous raw data to provide reliable and precise positions, thanks to the diversity obtained.
  Figure 1 shows the UML class diagram of the possible components that are defined in the proposed architecture, needed for considering the previous requirement. As we can see, we have *Nodes* of two types: *Anchor* (with fixed and known positions) or *Mobile* (to be located). The *Networks* are groups of several *Anchor* nodes (at least one) to form a common WSN. Finally, the *Target* devices are virtual devices with an identifier that aggregate several *Mobile* nodes (*i.e.*, different technologies) and/or generic *Sensors* which can be jointly located with only one system query. As we can see, target nodes can consider multiple technologies, providing diversity that can be exploited by the algorithms.
- **Multiple coordinate system and map-aware application support:** to give support to the map-aware applications in indoor scenarios, the architecture must be able to work with elements and positions with coordinates relative to different maps, which possibly have different local coordinate systems. The local coordinate systems can be defined by an origin point, a scale, and optionally an associated blueprint bitmap. Moreover, the architecture should support a global coordinate system such as the one used by satellite location systems, and should be able to combine both kinds of coordinate system transparently for users.
- **Data Fusion:** the system must provide mechanisms for data fusion coming from different raw measurements (technologies) and/or positions obtained from different algorithms. On one hand, low-level raw measurement fusion could be considered inside the location algorithms (*i.e.*, Kalman or Particle Filters), since an algorithm can obtain data coming from several technologies (sensors) associated to the same target. And, on the other hand, optional high-level fusion of final estimated positions must be provided directly by the platform. In this way, depending on the LBS considered, client applications could switch between, or combine, different algorithm position estimations.
- **Protection and Security:** the system should support at least one mechanism for secure authentication of external users who want to interact with the system, and optional protection of the data interchanged with the system, for instance by some means of data encryption.
- **API:** the platform must define an API describing the access methods, to enable client applications to interface with the RTLS. This API will define a boundary with syntax and semantics that specifies the seamless interface between external modules and the platform. Since the cited standards ISO24730-1:2006 [24] and ISO19762-5:2008 [25] do not cover all the requirements of the proposed platform, it is not possible to follow them. However, the API platform is based on them using standardized names and abbreviations for the different actors, commands, modules, parameters, *etc.*
- **Off-line data:** it must be possible to get measurements and position estimations on-line in real time and also off-line. Therefore, it should be possible to get the latest measurements or positions available or those from specific instants of time (off-line or historical data). Off-line data makes it possible to test and compare algorithms and client applications with a common set of data, and obtain repeatable results which are critical for research purposes.
- **Easy-to-Use:** users without special skills should be able to access the system to perform any of the following tasks:
  – Registration of WSNs, building blueprints, anchor networks, mobile nodes, generic sensors, and insertion of raw measurements into the system. This information will provide an abstraction of the hardware, offering users without hardware skills the possibility to obtain real measurements and sensor data without effort.
  – Obtaining measurements and inserting position estimation information by users who design location and tracking algorithms. They provide positioning data to end-user client applications which do not need special skills in how the location algorithms are implemented, optimized or obtain measurements.
  – Obtaining position information by end-user client applications in real time, for several mobile nodes, sensors, or groups of them, which can be implemented by using different WSN hardware technologies. These users do not need to know how and from which WSNs measurements were taken, nor from which location or tracking algorithms were these positions generated.

## 3. Logic View

**Figure 2.**
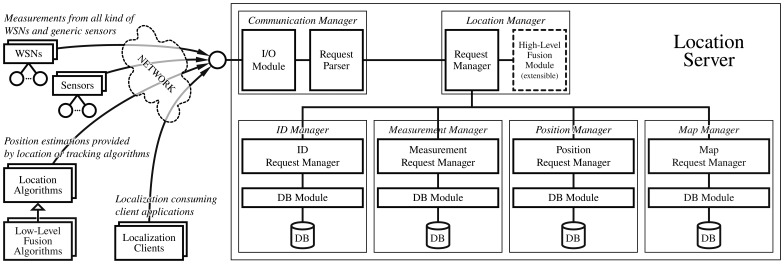
Proposed architecture for Hybrid Real-Time Location Systems.

Figure 2 shows the components that make up the proposed client-server architecture as well as the connections among the components in terms of a logic view:

- **Location Server:** the main component of the hybrid location system, which is composed of several modules for storing measurements and positioning information obtained from external WSNs, sensors, and location algorithms. It allows external actors to insert and retrieve in a flexible way all manner of location information. As a server, it offers an input/output interface to allow remote connections.
- **WSNs and Sensors:** remote client applications that can be deployed in distributed hosts, and which provide all kinds of measurements (as shown in Figure 1, RSS, TOA, TDOA, AOA or distance) and generic sensing data (*i.e.*, motion detection, acceleration, turning, inclination, *etc.*) to other actors such as Location *Algorithms*.
- **Location Algorithms:** client applications responsible for obtaining available measurements and sensing information from the *Location Server* to estimate new positions. Then, they can store the new positioning information inside the system. *Low-Level Fusion Algorithms* (*i.e.*, Kalman and Particle filters) are supported as specialized *Location Algorithms* which would retrieve several kinds of measurements from different WSNs (instead of from only one), accessing the *Location server.*
- **Localization Clients:** client applications that consume positioning information previously generated and filtered for one or more location algorithms, with the option of high-level position data fusion. They can also query for measurements if they need them for a specific purpose.

### 3.1. Location Server

Figure 2 shows a component diagram of the *Location Server* architecture. It is composed of the following subsystems:

- **Communication Manager:** responsible for communications with the server to attend external clients, parsing the received requests and generating appropriate responses. Internally, it is made up of an *Input-Output Module* in charge of the data transmission from or to an external network, and a *Request Parser* module in charge of processing query messages and wrapping output messages. As we will show in the following sections, these messages are well defined, based on the JavaScript Object Notation (JSON) standard. JSON is a lightweight text-based open standard designed for human-readable data interchange, much more compact and readable than other data formats such as XML [37] and YAML [38]. The greater speed and reduced computational complexity of JSON *versus* other formats has led the main API developments to increase their use of JSON from 6% in 2009 to 20% in 2011, as the only supported formatting system [39]. Seeing the growing inclination of developers and API makers alike for JSON, many well-known companies and Internet services such as Tumbler, Twitter, Facebook, OpenSocial, Google, Digg, Urban Airship, Twilio, *etc.* have decided to eliminate the XML support (when possible) and devote their energies to migrating to a good implementation of JSON [39–45]. Currently there are several implementations of JSON available (*i.e.*, Jackson [46], Jsonlib [47], Google Gson [48], *etc.*), which even offer cache mechanisms to guarantee maximum performance. Some benchmarks are available in [49–52]. Therefore, we decided to combine JSON with standard TCP sockets to guarantee very low in-system overheads due to message data processing, and to allow a very low latency. In this way we allow any client (even lightweight clients using micro-controllers), using any kind of programming language, to be able to send and receive data with the *Location Server*.
- **Location Manager:** its mission is to analyze all the queries that come into the *Location Server* to find a suitable answer in the server by delegating in the *Position Manager, Sensor Manager* or *ID Manager* subsystems. The next element is the *High-Level Fusion Module*. It is responsible for creating fusion data based on the available position estimations from any technology that have been generated by any *Location Algorithm*. This module is easily extensible by a programmer by registering a new fusion module implementation in the system. Adding a fusion method key to an appropriate configuration file and the implementation class should be enough to add a new high-level fusion method to the architecture.
- **ID Manager:** its mission is to store the existing relationships among the *target* devices to be located and the *mobile* nodes and *sensors* assigned to it (see Figure 1 for more details about the relationships allowed in the system). This subsystem manages the associations and disassociations among the target devices, mobile nodes and generic sensors, and a log of these associations for future use.
- **Measurements Manager:** receives and stores measurements from WSNs and sensors, and attends queries from external *Location Algorithms and Localization Clients*.
- **Position Manager:** its mission is to receive and provide positioning data. Several *Location Algorithms* can store estimated positions in the system using this module, while external *Localization Clients* can query these data at any time. The stored positioning data is associated with an identifier of the *target* device or single *mobile* node or *sensor* from where it was measured, and an estimation of its accuracy, which is calculated by means of different aspects such as the kind of technology, the number of sensors available, the number of measurements per time, *etc.* The associated identifier can be a single mobile device, although more sophisticated *target* devices are managed by the *ID Manager*.
- **Map Manager:** receives and stores coordinate system information associated to several maps. Map-aware elements such as the anchor nodes or the position estimations generated by the *Location Algorithms* (or a specific *Low-Level Fusion Algorithm*) are relative to a particular map registered in the system. The different clients can register new_maps, given an origin coordinate, a map scale and optionally a map bitmap (usually the blueprints of a floor of a building for an indoor scenario).

As shown in Figure 2, some subsystems are connected to different databases, which can be implemented in the same or in several physical databases. Also note that the *ID Manager, Measurement Manager, Position Manager and Map Manager* use the following two modules: a *Request Manager* to parse the queries and encapsulate the result data, and a *DB Module* to obtain and store information from or into the appropriate database, abstracting and isolating access to it.

When isolating each part within the *Location Server* as we have done, using different modules and layers, we are able to correctly maintain and extend each part in the future. Therefore, we can give a proper support to future application requirements, or adapt the system to specific project constraints with little effort.

### 3.2. High-Level Fusion Module

As we have explained in Section 2, multiple sensor technologies can be associated to a virtual target device that can be used for its location. With the proposed location system, we can not only query the position estimation of a multi-technology target device with one system call, moreover we can combine all or a subset of technologies grouped by a target device with one system call.

The proposed high-level fusion module of the architecture can combine or select different position estimations available in the system, which were inserted by several location algorithms and which are associated to several sensor networks. This module can automatically switch or combine the available position estimations, and provide an end-user client application with an improved position estimation, also automatically.

The main advantage of the high-level fusion module of the proposed architecture is its extensibility. A software programmer can add a new implementation for this fusion module to the existing ones, adding new capabilities to the system in terms of data fusion. The new data fusion method should be uniquely identified by a string key, which should be added to a configuration file, in addition to other parameters about the path of the new implementation class files, *etc.*

The proposed architecture message system that will be discussed in Section 4 will show how to ask the system for a position estimation, using or not the data fusion capability.

To facilitate the workload of the data-fusion method programmer, the new position estimation message defined in the system (*new position* message) requires an input position accuracy (*position.acc*) parameter. It is thus mandatory for all the location algorithms to insert an accuracy estimation (in meters) for every position estimation inserted into the system. It is a task of the location system designer to provide this parameter as accurately as possible, through the *new position* message. Typically based on the specific characteristics of the indoor scenario, the number of anchor nodes available, number of anchors detecting a mobile node, the kind of sensor technology (based on RSS, TOA, *etc.*), and maybe on an estimation of the variance of the signal level fluctuations, the location algorithm designer should provide the system a dynamic changing *position.acc* value.

As an example, we have considered three different data fusion techniques to illustrate how the proposed *high-level fusion module* works, and how it can be extended. Some of these methods take advantage of the *position.acc* information obtained from the location algorithm estimations, and by means of different techniques for combining or selecting the available position estimations provide filtered position estimations. The three methods implemented are based on classic combination and selection algorithms, typically used to increase the capacity or to stabilize the received signals when using multiple antennas. Note that in this paper we use the same names as those used in the traditional algorithms, but we have adapted them to our location architecture, based on the *position.acc* parameter and the position estimations. In the results Section 6 we will show the conditions where we gain from using high-level data fusion, compared with the case where we use separate individual WSN technologies.

#### 3.2.1. Combination Methods

- **EGC (Equal Gain Combiner):** identified as EGC in the system, this combination method is very simple as it gives equal weight to all the position estimations from the available technologies associated with a single device. It produces the average of all the position estimations, following the equation:
  (1) $$pos_{\text{EGC}}=\frac{1}{N}\sum_{i=1}^{N}pos_{i}$$
  where *N* is the number of available location algorithms which are associated to a device, and *pos_i_* is the position given by the *i*-th location algorithm. This method does not take into account the *position.acc* parameter.
- **MRC (Maximum Ratio Combiner):** identified as MRC. Unlike the EGC, this method weighs each position estimation depending on its quality, given by the *position.acc* parameter. That is, it gives more weight to the algorithms with the smaller *position.acc*, and is calculated by means of the following expression:
  (2) $$\begin{array}{cc} pos_{\text{MGC}} & =\sum_{i=1}^{N}\omega_{i}pos_{i},\\ \omega_{i} & =1-\frac{\text{position}.acc_{i}}{\sum_{j=1}^{N}\text{possition}.acc_{j}} \end{array}$$
  Note that in the event of there being only one position available (*N* = 1), the result is directly this position.

#### 3.2.2. Selection Methods

- **SC (Selection Combiner):** identified as SC, this method consists in sorting the available position estimations by means of their associated *position.acc* from lowest to highest value, choosing only one value. The goal of this method is to achieve the best position estimation at each instant, without considering the location accuracy fluctuations. In our system we have considered the position estimation associated to the lowest valued *position.ace.*

### 3.3. Location Algorithms and Low-Level Fusion Algorithms Support

As shown in Figure 2 any arbitrary location or more sophisticated tracking algorithm is supported. The typical workflow of the location algorithms is explained in detail in subsection 4.3 but basically, they are periodically fed with measurements from a particular WSN, which are stored in the *Location Server*, and they then generate new position estimations in real-time (which are sent back to the Location Server for future use by any *Locatization Client* of other *Location Algorithms, etc.*).

The special tracking algorithms which involve a low-level fusion of raw data from multiple WSN are specialized versions of a *Location Algorithm*, supported in this architecture by the gathering of measurements from several WSNs and sensors through the *Location Server.* The mechanism for obtaining measurements is the same. The multi-threaded implementation of the *Communication Manager* allows for concurrent connections to the *Location Server* and permits the low-level fusion algorithms to gather the sensor data asynchronously and concurrently at any time, so that they have time to process and merge the data as needed.

Therefore, all kinds of Bayesian filtering algorithm with different observation models are supported within this architecture.

### 3.4. Protection and Security

We considered the OpenID [53] open standard as the most convenient manner of authenticating users with the *Location Server*. It is a highly mature and safe solution, which can be extended in the future. OpenID authentication is now used and provided by several large websites, such as AOL, BBC, Google, Yahoo!, IBM, MySpace, LiveJournal, Facebook, Vkontacte, Steam, Orange, PayPal and VeriSign.

Regarding the confidentiality of data exchanged between the different clients and the *Location Server*, we can optionally add cryptographic support by using security protocols such as Secure Sockets Layer (SSL) [54] or Transport Layer Security (TLS) [55]. These security mechanisms are optional and independent of the rest of the proposed architecture, and they are only implemented for high-level clients with high security requirements. Thin clients such as the ones implemented over a WSN hardware do not usually require such high security which, on the other hand, would increase the network overheads.

## 4. Runtime View

After describing the elements (and interconnections) of the proposed architecture in Section 3, in this section we will explain a runtime view which represents the workflow of the different processes needed by the proposed architecture supporting the capabilities shown in Section 2.

These processes are divided into the following four main groups, according to the person responsible for carrying each process. All the processes are considered independent:

- **Network Administration and Measuring Processes:** a network installer has to physically deploy a WSN (of anchor nodes) at an indoor or outdoor scenario, from which to take physical measurements. Therefore, a user registers the *network* elements (anchors), *mobiles* and *generic sensors*, all of them with an associated identifier, and the anchors with their real and fixed positions relative to a *map* coordinate system. After that, several external software applications should continuously take measurements from the deployed networks by directly accessing the nodes hardware. They then insert these measurements into the system by creating standardized messages and sending them through a network to the *Location Server*. In this section we will explain in detail the set of standardized commands supported by the system.
- **Target Device Administration:** the same network installer or another user can register virtual *target* devices, which are associated to several *mobile* nodes and/or generic *sensors* used for localization. These targets, and consequently the several elements associated to them, can be queried by other users (in the following steps) with a single system call.
- **Position Estimation using Location Algorithms:** a researcher or location algorithm developer can continuously take measurements from the system, without taking care about how the WSN networks were deployed, or how the measurements were “extracted” from the hardware. These users can implement several variants of a location algorithm which are fed with real measurements, from the same or from different WSNs. Specialized versions of *Location Algorithms* can perform low-level data fusion by retrieving measurements from several *networks* and several *sensors* at a time. After that, they can perform a tracking step, and finally return a position estimation to the *Location Server*. As always, the measurements and positions can be easily obtained from the system or inserted into the system by using standardized commands.
- **Localization Clients:** finally, several end-user client applications can access the system in real time to obtain position estimations from several technologies, optionally using a high-level fusion method registered in the system.

**Figure 3.**
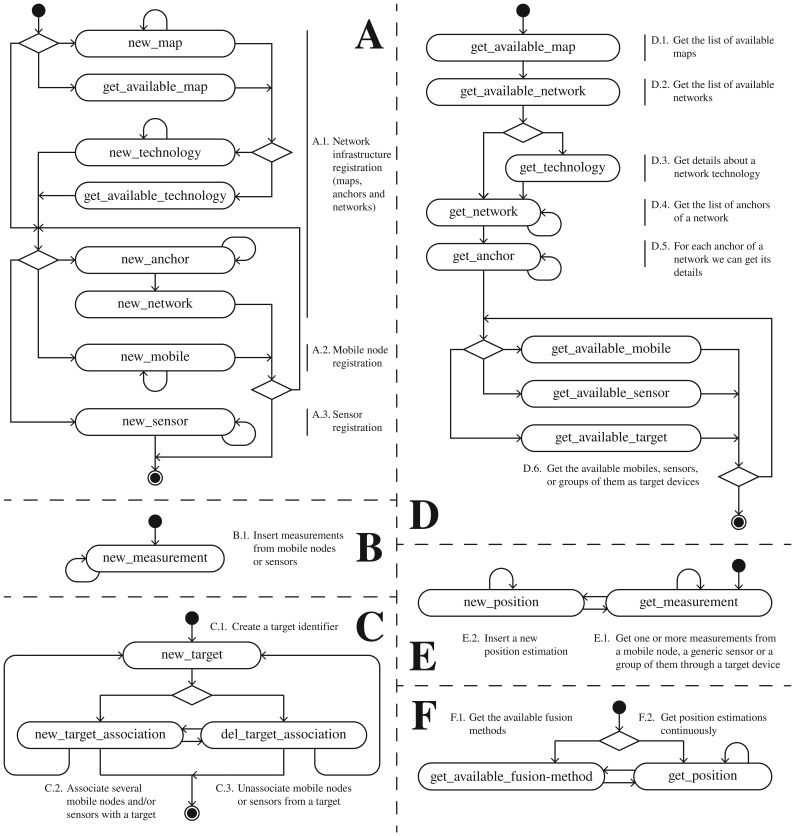
Workflow of the proposed system divided into six groups of messages: (**A**) Infrastructure (maps, anchor and network), mobile node and sensor registration process; (**B**) New measurements from mobiles or sensors; (**C**) Target administration with association to mobiles and/or sensors; (**D**) Location algorithm initialization steps to know the elements available; (**E**) Position estimation process where, a location algorithm estimates new positions after reading measurements; (**F**) The localization clients continuously get position estimations, optionally filtering and merging positioning data.

In the rest of this section we will decompose the previous main groups of processes, and explain them in depth. Figure 3 shows six groups of processes. As we will explain later, the reason for dividing them into these subgroups is because some of these processes need only be performed once, for instance for initialization purposes.

### 4.1. Network Administration and Measuring Processes

These processes correspond to the workflow of the group of commands A and B shown in Figure 3, and they are explained below:

#### A.1. Network Infrastructure Registration

First, a network installer would physically deploy the anchor nodes of a network (group of nodes with fixed positions) at an indoor or outdoor scenario. Then, he has the option to make the local coordinate system of several floors of a building with their associated blueprints available for future use. He can use the new_map command for this purpose, or obtain the maps already uploaded into the system by using the get_available_maps command. After that, if this has not been done before, he registers the kind of node technology (*i.e.*, ZigBee, based on RSS measurements or UWB, based on TOA measurements), using the command new_technology. This step is performed only once for a specific kind of hardware technology of a network (*i.e.*, ZigBee infrastructure based on MicaZ [56] nodes working at 2.4 GHz in channel 15, with a specific PAN identifier, *etc.*). This means that when using another ZigBee platform, for instance based on Arduino [57] plus XBee [58] nodes, or with the same hardware but configured in another radio channel with a different PAN ID, *etc.*, which is not interoperable with the previous one, the new technology should be registered as a new kind of technology in the system, with a different identifier. He also has the option to retrieve the previously registered WSN or sensor technologies by using get_available_technology.

Once the technologies have been registered, the network installer has several alternatives. He usually continues inserting the WSN anchor nodes with their fixed positions into the system (*i.e.*, ten anchors for a desired room, calling the new_anchor command ten times). The anchors are associated to a specific technology, which guarantees a type and format of measurements for the system in the future.

Then, it aggregates all these anchors to a same network identifier (which represents a unique WSN) using the new_network command. Thus, in the future all of these grouped anchors can be accessed at the same time by a location algorithm. Note that an anchor can only be associated to a single network and that its coordinates are relative to the local coordinate system defined for a specific map (floor or part of a building or other scenario with an origin coordinate, a scale (in pixels/meter) and an optional associated blueprint bitmap image). An example of a new_map JSON message is shown in the next section.

As an alternative at the third decision point, the network installer could skip the anchor and network registration, and pass directly to the following mobile node or sensor registration. Note that some previously registered *technologies* only involve generic *sensors* which are not associated to any registered network (*i.e.*, inertial sensor, fingerprinting scan point, *etc.*). See group A of messages in Figure 3 for more details.

This registration process can be repeated for a different kind of node platform and networks as many times as needed. Other users who have already implemented some location algorithms and tested them with simulated measurements can now test their algorithms with real measurements, without having to be skilled in either hardware deployment or low-level programming of access to the WSN hardware in order to extract physical measurements.

#### A.2. Mobile Node Registration

The network installer also registers new mobile nodes in the system for use by other users with no hardware skills. He uses the new_mobile command.

#### A.3. Generic Sensor Registration

The final registration step is for generic sensors such as accelerometers, gyroscopes, digital compass, *etc.*, which can give localization support to a tracking algorithm, for instance. They must be registered at this step using the new_sensor command. Note that a customized kind of technology must have previously been registered in step A.1 for each generic sensor. For instance, for a 3D accelerometer we would require three float values associated to each measurement, representing the acceleration on the three axes. Each sensor measurement format is unique and can be custom defined as shown in Figure 1, as any combination of data types supported by the system. The system will use this kind of custom technologies so defined to parse and verify the format of new measurements to be inserted into the system in the future.

#### B.1-2. Measuring Process

The next two measuring process steps can be performed by the same network installer as in the previous registration steps, or by another user who is familiar with the deployed hardware platform and who knows how to extract physical measurements from the hardware. This user should implement a piece of software that continuously accesses one or several anchor nodes at low-level (which can be quite sophisticated depending on the kind of technology) to be able to communicate or somehow detect several mobile nodes. This software finally extracts physical parameter information like RSS, TOA, AOA, *etc.*, or generic sensor data (for generic sensors such as accelerometers, gyroscopes, *etc.*). It should use the new_measurement command to insert new measurements into the system, and make them available for the other users (*i.e.*, the location algorithm designer or an end-user location application).

### 4.2. Target Device Administration

As explained in Section 2 we can group and identify several *mobile* nodes and generic *sensors* by a single and unique *target* device identifier. In this step, somebody in charge of the device administration can create new virtual target devices and associate or disassociate mobile nodes or generic sensors with or from it. The new_target command can be used to register a new target device identifier in the system, and then the new_target_association and del_target_association commands can beused to add or to delete mobile nodes and sensors to the target device. This process corresponds to the workflow and the group of commands C shown in Figure 3.

### 4.3. Position Estimation using Location Algorithms

The researcher or user who implements a location algorithm is the next actor in the system. He would typically be a user who has already implemented a more or less complex location or tracking algorithm, and who probably uses a simulation environment such as MATLAB and makes use of simulation measurements to test his algorithms. This user can now feed his algorithm using real measurements from one or several WSNs, with the advantage that he does not have to spend any time deploying a WSN or accessing the hardware. He can directly query which *technologies* are available (registered) in the system, where the *networks* (groups of *anchors*) are physically deployed (relative to a *map*), with which *technology* a particular *network* (or kind of *sensor*) is implemented, which *mobile* nodes and *sensors* are available for experiments, *etc.* Finally, he can query for several on-line or off-line measurements from several WSNs. All these tasks can be easily performed using standard commands as always, with no need for advanced skills in hardware or software implementation. In Section 5 we will explain how a researcher could connect the *Location Server* to take real measurements.

This process corresponds to the workflow and the groups of commands D and E shown in Figure 3. The first part (group D of commands), as an initialization step, is only performed once to know which *maps, technologies* and *networks* are available, where the *anchor* nodes are deployed, and which *target* devices, *mobile* nodes or *sensors* are available for testing. Remember that the networks and other nodes were probably deployed and registered by another user. Taking this information into account, the researcher can now configure his location algorithm parameters, and enter the place where the network is deployed (*i.e.*, in a building) to make measurement studies and test his algorithms with real measurements.

Once his algorithms have been initialized with the information for a specific network, the researcher should start getting measurements from the system in a continuous fashion, and then generate position estimations based on them. This second group of commands is depicted as E in Figure 3. Finally, he will register these positions in the system, making them available to other *Localization Clients* who do not have skills in location algorithms and only want to obtain final position estimations.

### 4.4. Localization Clients

Finally, a localization-consuming client application wants to remotely query the position estimation of a target device (that can be multi-technology) or of a single mobile node or sensor. This user does not need to have any skills in how the WSN was deployed, how the measurements were extracted from the hardware or how the positions were estimated by one or several location algorithms.

This process corresponds to the workflow and group of commands F shown in Figure 3. Firstly, the user can optionally ask for the available high-level fusion methods registered in the system, to later obtain position estimations using data fusion. Alternatively, he can directly and continuously query the system for position estimations for a specific target device, mobile node or generic sensor. Note that he could also perform some initialization steps as in the group of commands D, to know the maps, networks, targets, mobile nodes and sensors available.

**Figure 4.**
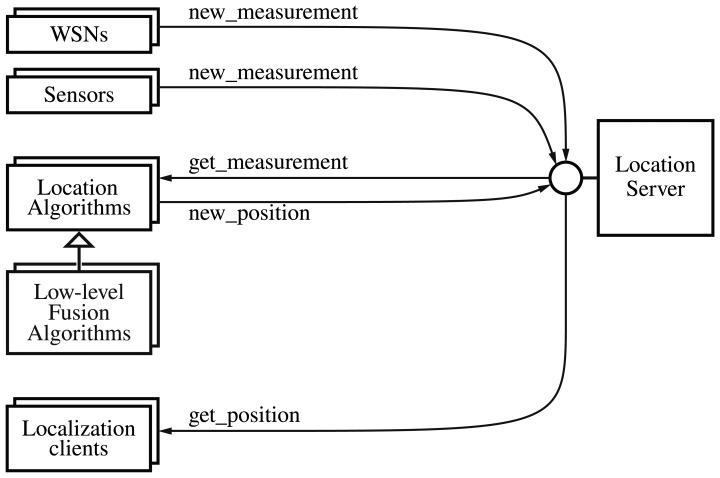
Once the network infrastructure is registered, and the location algorithms and clients configured, these actors continuously use the same type of messages to save and retrieve data with the system.

To summarize this section, Figure 4 shows the set of the messages that all the actors would continuously use after their initialization steps.

Note that we do not show other messages to modify and delete the information available in the *Location Server.* Commands such as set_map, set_technology, set_anchor, set_network, set_mobile, set_sensor, del_map, del_technology, del_anchor, del_network, del_mobile, del_sensor, del_target, del_measurement and del_position are also available to privileged users. We support not only a global coordinate system for anchors and position estimations but also relative position coordinates inside a building, covering all the common scenarios.

## 5. Implementation Example Case Studies

In this section we will explain two implementation example case studies to better understand the capabilities of the system regarding the fusion of multiple technologies, the mixing of mobile nodes and generic sensors, how to define new kind of sensor technologies, *etc.*

### 5.1. ZigBee and UWB Networks, plus an Inertial Sensor (Accelerometer)

In the first case, we have chosen the ZigBee and UWB technologies plus some inertial sensors (acting as generic sensors) to better explain how the commands described in the previous Section 4 are formatted, in the case of having a multi-technology target device to be located. Moreover, we will make it clear how to call the system to obtain high-level fusion position estimations in this case.

The ZigBee network could be implemented with MicaZ [56] nodes working at 2.4 GHz. In this example we will have four anchor nodes zb_Al to zb_A4 and one ZigBee mobile node zb_Ml.

The UWB network could be implemented with the PLUS platform of TimeDomain [32]. In this example we will have four anchor nodes (UWB readers uwb_Al to uwb_A4) and one UWB mobile node (UWB tag uwb_Ml).

Additionally, we have decided to take the measurements from the accelerometer ( accel_l) integrated in a smartphone to illustrate how the measurements of any generic *sensor* could be formatted and processed.

**Figure 5.**
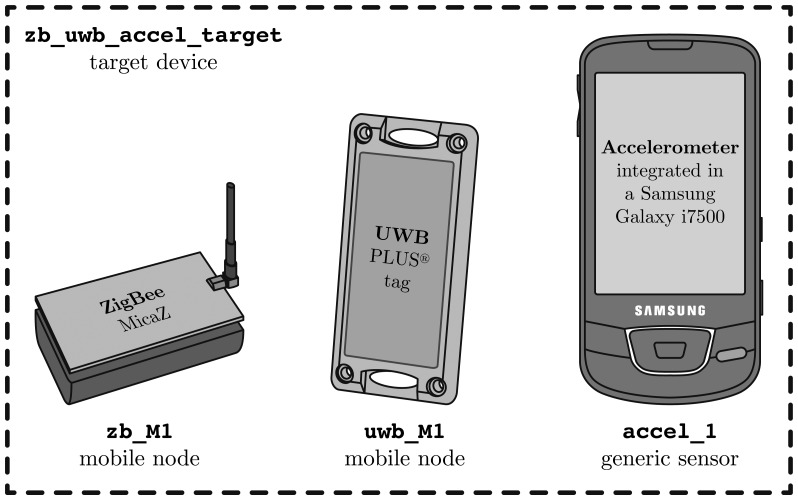
The zb_uwb_accel_target identifier represents a virtual target device, and it is associated to the zb_Ml and uwb_Ml mobile devices and to the accel_l generic sensor.

The ZigBee and UWB mobile nodes, plus the accelerometer, will be associated together by a unique *target* device identifier, in order to be conveniently located with a single system call. Figure 5 shows how we associate a unique identifier to the virtual target device associated to both mobile nodes and the sensor. Note that all these pieces of hardware should be attached and moved together in order to be tracked correctly. In a real case, they would all be integrated in a multi-technology portable device.

In order to save space in this section, we will usually refrain from showing some kinds of messages twice, for the cases where the ZigBee and UWB messages are somehow identically formatted. We will also usually refrain from showing the reply commands obtained from the system, when they are simply OK acknowledgement messages, as they actually are in most of the cases. We always assume that there will be no communication problems with the location server.

Next, in this section we will explain the four kind of processes introduced in Section 4, applied to the example case study of using ZigBee, UWB and an accelerometer, by using similar flowchart diagrams but with specific JSON messages for each entity.

**Figure 6.**
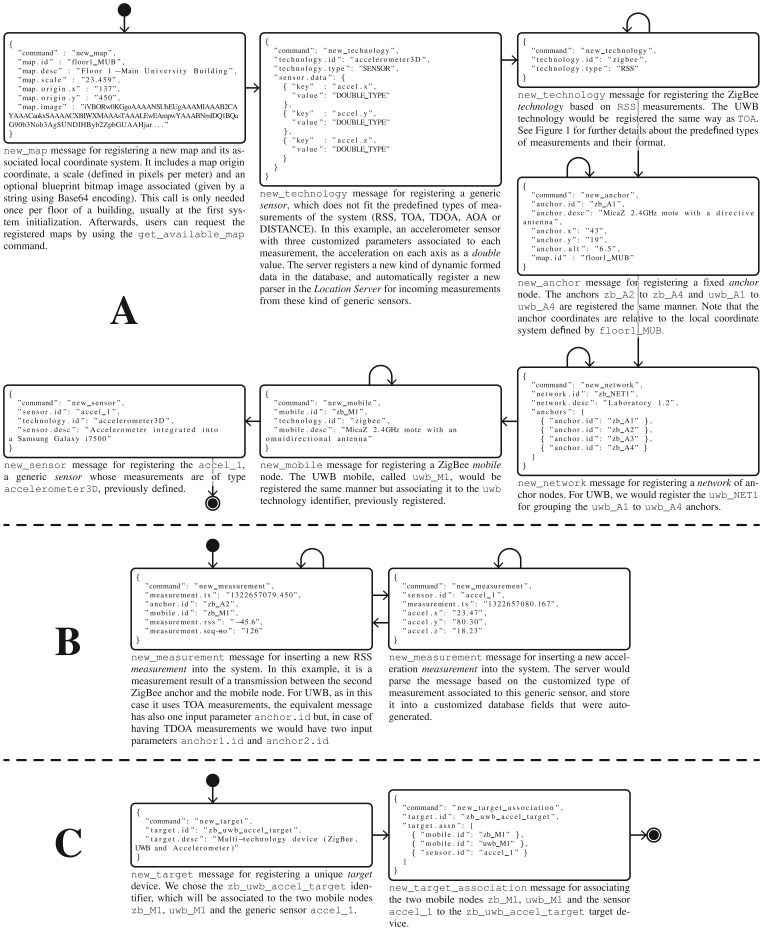
Groups of messages A, B and C.

As in Figure 3 in Section 4, groups A and B of messages in Figure 6 show the network administration and measuring processes in detail. For the measuring process we assume that we have already implemented three pieces of software for obtaining measurements from the ZigBee, UWB and accelerometer hardware. On the one hand, a client collects RSS data coming to a PC by USB port, from a ZigBee gateway (or a sniffer). On the other, another client collects TOA measurements from the TimeDomain UWB platform. With the software API provided by TimeDomain, it is quite straightforward to implement a client that takes raw TOA measurements from the hardware and optionally transform them into TDOA measurements (when needed, grouping the anchor TOA messages in pairs). These two pieces of software that abstract the communications with the WSN hardware have to insert the new RSS and TOA measurements (when they are available) into the *Location Server*, by using the new_measurement command (as shown in the first message of group B in Figure 6). Finally, a small piece of software, implemented in Android for example, could collect the inertial information from its accelerometer by a predefined sampling period of time, and periodically send it to the *Location* Server by also using the new_measurement command (with different parameters).

The target device administration is explained by group C of messages in Figure 6.

**Figure 7.**
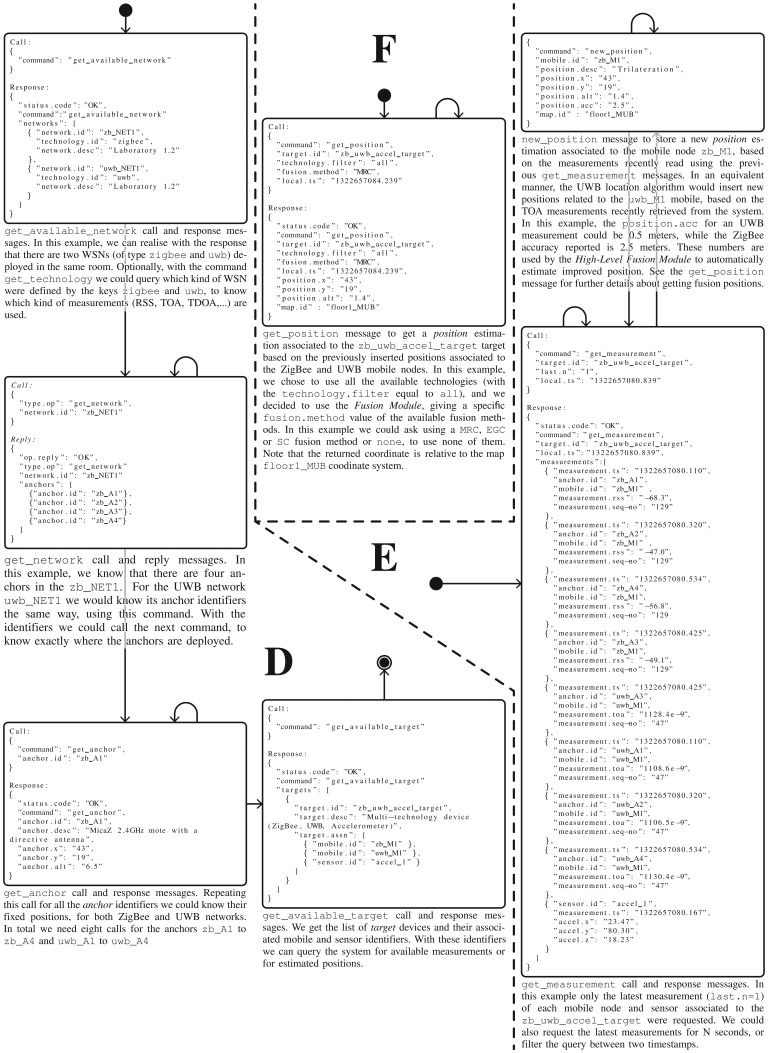
Groups of messages D, E and F.

The position estimation using location algorithms is shown in group D of messages in Figure 7. We show several commands for the initialization of the location algorithms, while in group E of messages we illustrate the loop process of obtaining measurements from the system (for a desired target, mobile node or sensor) and inserting new position estimations. In this example, two location algorithms should be implemented in order to process the RSS and TOA measurements separately. Usually the location algorithms are implemented by a researcher using a high-level simulation environment such as MATLAB, and he only has to add some functions to send and receive JSON messages using TCP/IP sockets. This is quite straightforward using a Java client for processing JSON messages, as MATLAB is completely integrated with the Java Virtual Machine (JVM).

As explained in Section 2 we wanted a highly flexible system for obtaining both measurements and positions. Our implementation allows to obtain both on-line and off-line measurements in real time, asking in different ways:

- We can ask for a list of the latest N available measurements stored in the server DB.
- Asking for the available measurements in the last S seconds, starting the count from the time when the call message is received by the server. Note that we can retrieve a greater or smaller number of measurements, depending on the sampling period of the WSN measuring client.
- Or we can ask for off-line measurements registered in the past. This is a flexible manner to test location algorithms using the same campaign of measurements in each case, to produce repeatable results. The simulation results that are explained in Section 6 are obtained using this strategy. Note that we can insert both real and simulated measurements to test a location algorithm by sending new_measurement commands through the network. The mechanism for inserting the measurements into the system is the same in both cases, using the new_measurement command filled in with simulated or real data. Therefore, for testing purposes we can also take advantage of this feature, and simulate thousands of nodes and large numbers of location algorithms accessing the system at the same time. This allow us to perform stress tests, measure latency *versus* load, check the performance of disk access to the DB under certain controlled conditions, *etc.*

Finally, the *Localization Clients* can use the get_position command to obtain position estimations from a remote computer or device, via LAN or Internet connection. As shown in group F of messages in Figure 7, and similarly to the case of get_measurement, they can request on-line and off-line position estimations: the latest N known positions, the latest positions stored for S seconds starting from the present, or a list of positions between two epoch timestamps.

Moreover, they can filter by technology when asking for a target device position, to use all or a subset of the mobile nodes and sensors associated to a target device. Finally, they can optionally make use of a high-level position fusion method implemented in the *Location Server*, such as the EGC, MRC or SC proposed in this article for exemplifying purposes. See Section 3.2 for more details about the fusion methods implemented.

### 5.2. WiFi Fingerprinting

In this second example case study, we will show how to apply our architecture in a real indoor scenario when using RSS measurements and a fingerprinting location algorithm [11,59].

We take advantage of the WiFi access points already deployed in a building acting as beacon transmitters. We use a WiFi tag that during the *off-line* or *calibration* phase acting as an access point (AP) scanner, measuring at different locations of a building. The Received Signal Strength Indicator (RSSI) from several APs is measured at chosen locations, called *scan points* (SP) *or calibration points*. These measurements are called *fingerprints* of the SPs and they are part of a calibrated *radio map*[11,60–62]. Then, during an *on-line or location estimation* phase of the algorithm, the WiFi tag acts as a target sensor, detecting the nearby APs while it moves. For simplicity, in this example we assume that the state of the target includes only its location (*x* and *y* coordinates), and we associate the SP to a zone identifier of the building for convenience.

**Figure 8.**
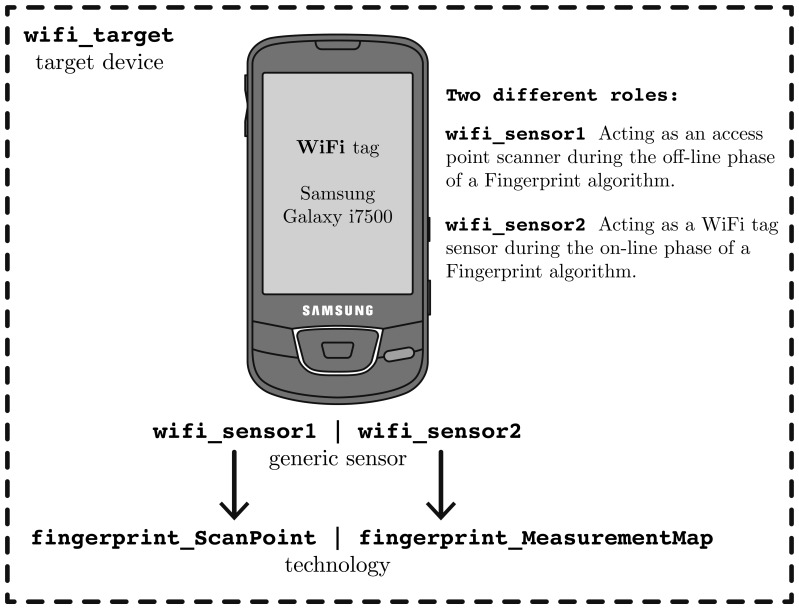
The wifi_target represents a target device associated to the wifi_sensor1 or wifi_sensor2 implemented with the same physical smartphone device, in the example.

We have used an Android smartphone with integrated WiFi connectivity to implement both roles during the off-line and on-line phases, as shown in Figure 8.

First we specifically define the data structures for storing the measurements and the fingerprints associated to each SP. Conceptually we have chosen the most complete way to represent this information as raw lists of RSS measurements associated to an arbitrary number of surrounding APs. This way the more or less complex fingerprint algorithm can use not only the mean [11], mean and variance [59], number of available RSS measurements associated to an AP identifier, and also the whole list of measurements to define a histogram [63].

**Figure 9.**
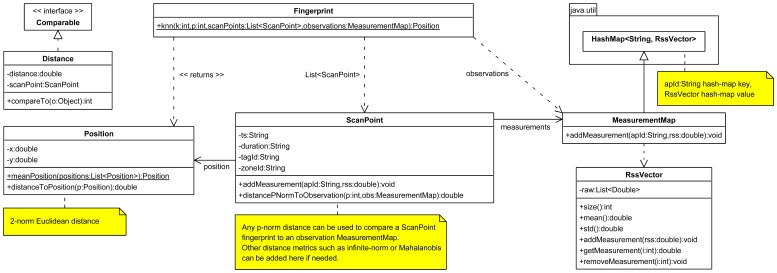
UML class diagram representing how to implement any Fingerprinting algorithm in any language (e.g., a KNN programmed in Java).

Figure 9 shows a UML class diagram representing the data structures needed. On the other hand, we have defined a RssVector class which makes it possible to store an arbitrary long RSS list of measurements, which is associated to a specific AP by using a hash-map MeasurementMap. We can add new RSS measurements to a MeasurementMap for an specific pair (apId-rss), by calling its addMeasurement(apId, rss) method. It first checks if there are previous measurements associated to the apId by searching in the list of available keys, and then it decides whether or not to call the addMeasurement(rss) method of its associated RssVector. Otherwise, it creates a new key-value entry in the MeasurementMap with the key apId associated to an empty RssVector.

On the other hand, a fingerprint associated to an SP is modelled with the ScanPoint class. The coordinates of the SP are stored in the position object (of kind Position). We also have some attributes such as ts (to store the timestamp of the beginning of the fingerprint), a duration (in seconds, to store how long we have measured in the said SP), a zoneId (zone identifier to associate the fingerprint to a specific zone of a building, for convenience), a tagId (tag identifier used for calibration) and, finally, a measurements attribute associated to a MeasurementMap hash map object which contains the whole fingerprint measurements.

Finally, the FingerPrint class has a static method to directly run the fingerprinting algorithm from any client without instantiating it. In this example we implemented a K-Nearest Neighbour (KNN) fingerprint algorithm [62,64] as it is a commonly used fingerprint algorithm in the literature. As we can see, with the k parameter we specify how many K “nearest” neighbours coordinates are used to average the position estimations. With a parameter p we choose the p-norm distance metric used to compare each ScanPoint fingerprint with an observation MeasurementMap. Finally we have to give as parameter the list of ScanPoint (of a specific radio map) and an observation MeasurementMap hash-map with the latest observed RSS measurements.

Following once again the workflow process explained in Section 4, we have to prepare the server for the available target and sensors, and register the new kind of sensor data that the WiFi sensors will send to the *Location Server*. In this case, we have two new kinds of sensor measurements to represent a fingerprint associated to an SP (during the off-line phase) and an observation from multiple APs (during the on-line phase).

**Figure 10.**
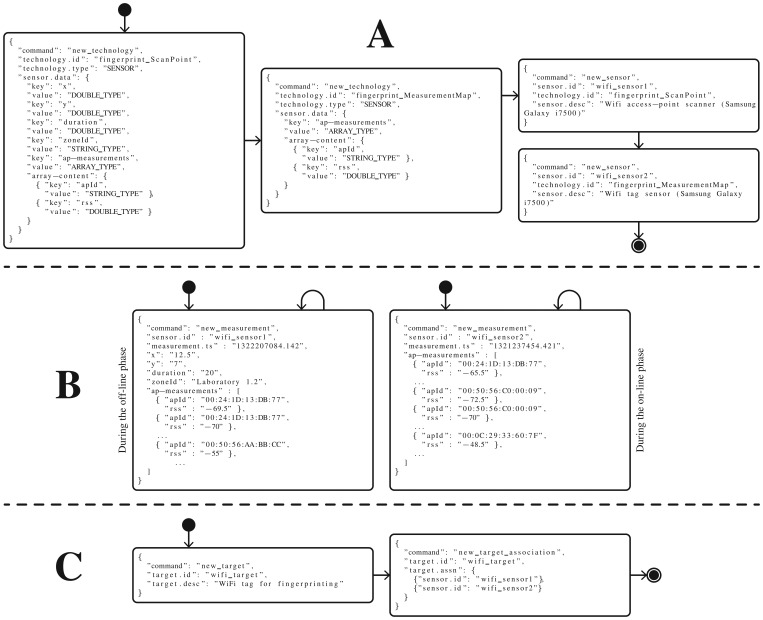
Groups of messages A, B and C of the fingerprint case study.

In Figure 10 we show a flowchart diagram with some of the custom defined JSON messages. During the network and target device administration processes we have to define the two kinds of *technologies*, then register the two WiFi sensors associated to each of the technologies, and finally associate both sensors to a unique target device for convenience, to facilitate the work of obtaining positions at a higher level for other clients. A localization client can easily ask for the wifi_target positions. During the measuring process we have to give measurements to the system with the new_measurement command: fingerprints during the off-line phase, and observations during the on-line phase, as shown in Figure 10.

After this point, we have to implement two pieces of software into our WiFi tag device (in our example an Android smartphone):

- To support the first role of the tag as an AP scanner during the off-line phase of the fingerprinting algorithm, we have to implement an application with a more or less complex Graphical User Interface (GUI) where we can fill the *position* coordinates (x,y) of the SP, the *duration* of scan, and a *zone identifier* from where we will scan (Another possibility would be to take advantage of the maps already registered in the system, retrieving all or some of them through the network using the get_available_map command, and performing the same kind of tasks: show the different scan point positions directly on each map in the GUI, allow the user to click on a relative coordinate of a map, etc.) After pressing a “Start” kind of button of the GUI, the application will call the WiFi API of Android to retrieve the surrounding AP measurements during few seconds, using the WiFi interface in monitor mode by detecting RSSI levels of the surrounding APs during a scan window on all or a subset of the WiFi channels. The application in this example can easily be implemented in Java (as Android uses Java) using the same data structures as defined in Figure 9. When the “Start” button is pressed we also start a timer and the application annotates the current system time as a UNIX timestamp. Once a timer exceeds the given *duration* time, the program will stop feeding the *ScanPoint* class with new measurements (pairs apId-rss), and it will send the whole fingerprint associated to the SP to the *Location Server* by using a single JSON message new_measurement, putting its own *sensor.id* to “wifi sensor1”, the timestamp to the current time of the system, and the remaining parameters according to the definition of fingerprint_ScanPoint technology. See Figure 10 for further details about the JSON messages. This process can be repeated as many times as needed, moving the WiFi tag to another position of the building, updating the current scanning coordinate on the GUI, pressing “Start” again, and so on.
- To support the second role of the tag as RSS scanner during the on-line phase of the fingerprinting algorithm, we have to implement a second application that could run in the background or not, which never stops sending measurements to the *Location Server*. In a non-ending loop it calls the WiFi API of Android to scan different channels and after a predefined period of time (*i.e.*, 2 s) it packages all the available observations to the server using the new_measurement command, putting its own *sensor.id* to “ wifi_sensor2”, the timestamp to the current time of the system, and the remaining parameters according to the definition of fingerprint_MeasurementMap technology.

On the other hand, a location algorithm researcher can execute a KNN fingerprinting algorithm (for example implemented in Matlab) to estimate positions of the WiFi target. First, he initializes it using several fingerprints associated to the SPs, *i.e.*, *fingerprint_ScanPoint* data, which can be retrieved from the *Location Server* using the get_measurement command. Then he gets real observations at a certain sampling speed, *i.e.*, *fingerprint_MeasurementMap* data, again using the *get_measurement* command. After each position estimation is calculated by the fingerprinting algorithm, he can optionally decide to filter the new position estimation based on previous position and a map cartography. Finally, he has to send each position estimation to the *Location Server* using the new_position command. This process is repeated continuously.

Finally, one or several *Localization Clients*, as in Section 5.1, can ask for the position estimations of the wifi_target and show the information in a GUI application, a webpage, or wherever they want.

## 6. Validation Results

Up to this point we have shown how different actors involved in a location system would interact with a *Location Server* by sending and receiving JSON messages through a LAN or Internet network, when we have different WSNs and devices to be located, different fusion methods available, *etc.*

In this section we present some validation results by considering virtual hardware nodes whose measurements will be simulated by software. Only the measurements are simulated based on propagation models and real scenario noisy conditions. The rest of the code is common for a real implementation, and we send and receive messages with the *Location Server* through a real network. Therefore, using the same software to work with real measurements is straightforward. Also note that with a simulated scenario we can perform stress tests with the server, evaluate the performance of a huge amount of concurrent connections, simulate many kinds of sensors available at the same time, various fusion methods, *etc.*

The simulated scenario consists of two ZigBee and UWB WSNs deployed in an indoor scenario. We will track the position of two mobile devices (a ZigBee node and a UWB tag) testing the three illustrative high-level fusion methods shown in Section 3.2. We make use of the LocationClient Java client partially introduced in the previous section, and from MATLAB we simulate a moving target device associated with the two mobile devices of the ZigBee and UWB networks, which moves around a large room where both networks were deployed. This Java client sends real messages through the network between a computer with Matlab (acting as a client) and another computer where we deployed the *Location Server* software plus a MySQL database. The server is implemented using Java language and it supports high-performance mechanisms as a pool of threads to attend concurrent TCP socket connections and a pool of connections to the MySQL database in order to support hundreds of concurrent requests from the network. In this way the system is scalable to support even thousands of concurrent requests.

We simulated the different actors of the location system:

- **The WSNs client:** Instead of getting real measurements from a hardware network, we will simulate RSS and ToA noisy measurements, based on noise levels that typically appear in real indoor scenarios [65]. Each *T_8_* = 0.5s we will have a new measurement when a mobile node is inside the coverage area of an anchor node of the same technology. On each iteration we will send each measurement to the server through the network using the new_measurement command, as would be done with real measurements obtained from a real WSN (when they are available).
- **Location Algorithm:** The second actor of the system is the location algorithm responsible for estimating new positions based on the measurements obtained from the system, and previously introduced by the WSNs clients. In this case we make use of the get_measurement and new_-position commands to retrieve measurements and store new positions in the server. We will explain the location algorithms used in more detail below.
- **Location client:** The last actor will track the positions generated by the location algorithms by using the get position command, querying the ZigBee and UWB nodes separately and together, using the three high-level fusion methods proposed.

**Figure 11.**
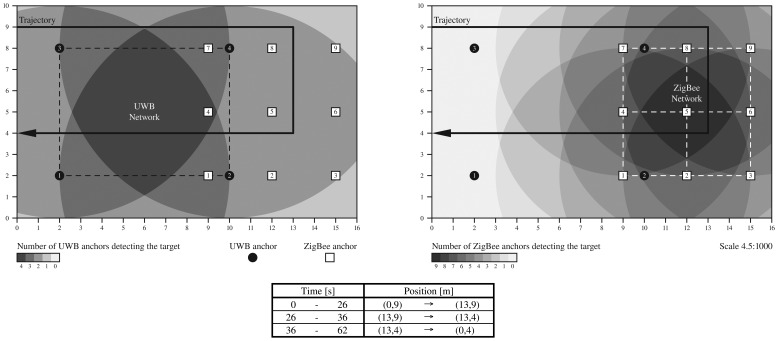
Simulated indoor scenario with two ZigBee and UWB networks deployed.

Figure 11 shows the indoor scenario where the two WSNs are deployed. There are four UWB anchors deployed on the left-hand side of the 16×10 meter scenario, with a simulated coverage of eight meters, and nine ZigBee anchors deployed on the right-hand side of the room, with a simulated coverage of six meters. With different grey scale colours we represent the coverage areas where we will be able to track the UWB and ZigBee nodes respectively. Note that we will need measurements from at least three anchors at the same time to be able to estimate a new position.

The networks are in partial overlap to simulate the effect of having coverage with only one of the networks, then with both, then again only with the second network, and so on. We will analyze the effect of tracking each mobile node separately, versus the use of both technologies together by means of the high-level fusion module available in the *Location Server*.

The trajectory is quite simple, going from one side of the room to the other, turning twice to the right and coming back again, crossing through the area of maximum coverage of both networks. At the bottom of Figure 11 we show the time spent in walking one round of the trajectory at a speed of 0.5 m/s. In total 62 s are needed to perform one round.

For the UWB network we assume we have TOA measurements affected by Gaussian noise with zero mean and 1 ns of standard deviation, while in the case of ZigBee we assume an exponential log-normal path-loss propagation model [66] with a Gaussian noise of zero mean and 3 dBm of standard deviation. The path-loss exponent was assumed as known and constant (equal to 1.8) throughout the simulation. Therefore, we assume that the received signal strength decays with the power of 1.8 each meter after a reference distance considered equal to 0.5 m.

Since the aim of this article is not to compare algorithm performance, we have considered the well-known Non-linear Least Squares algorithm [67] (we used the Nonlinear Least-Squares algorithm available in MATLAB using the lsqnonlin function of the *Optimization Toolbox*) for both technologies, ZigBee and UWB. With this algorithm we can minimize the sum of the squares of the distance errors to the anchors by minimizing the following objective function:

(3) $$\begin{array}{l} F(x,y)=\sum_{i=1}^{N}{\left({\bar{r}}_{i}-r_{i}\right)}^{2}=\sum_{i=1}^{N}f_{i}{\left(x,y\right)}^{2},\\ f_{i}(x,y)={\bar{r}}_{i}-r_{i}=\sqrt{{\left(x-x_{i}\right)}^{2}+{\left(y-y_{i}\right)}^{2}}-r_{i} \end{array}$$

where *r̅_i_* is the exact radio of the circumference centred in the i-th anchor, *r_i_* is the noisy radio of the i-th anchor, and (*x_i_, y_i_*) is the known position of the i-th anchor.

To be able to properly use the high-level fusion module of the *Location Server*, the location algorithm designer must provide an appropriate position accuracy (*position.acc*) value inside each new position estimation (as a parameter in each new_position command). After performing one survey round and observing the instant error for both ZigBee and UWB cases, we have chosen the following *position.acc* formulas for the simulations:

(4) $$\text{position}.{\text{acc}}_{\text{ZB}}=1+0.042{\left(\text{detecting}\_\text{anchors}-N\right)}^{2}$$

(5) $$\text{position}.{\text{acc}}_{\text{UWB}}=0.5$$

where *N* is the total amount of ZigBee anchors, and *detecting_anchors* is the number of ZigBee anchors detecting the mobile node. With the first quadratic formula, we have a maximum accuracy of 1 m when there are nine active ZigBee anchors, and a minimum accuracy of about 2.5 m when there are only three anchors covering the mobile node. The decision to choose an accuracy bound between 1 and 2.5 m is simply a possibility that the location algorithm designer should choose according to the scenario and available number of anchors. In this case a separation of 3 m between ZigBee anchors lets us choose a lower bound of about 2.5 m (when *detecting_anchors* = 3), while in the best case of having nine anchors detecting the target, uncertainty could reach the 1 m level (in this case the right hand side of equation 4 would become zero as *detecting_anchors* = 9 = N). Therefore, the constant 0.042 was chosen to reach the 2.5 m lower bound, and should be adjusted, or the whole position.acc*_ZB_* calculation could be replaced by another formula. For the UWB case, we fix the accuracy to 0.5 m in all cases, regardless of the number of UWB detecting anchors. Thus for instance, the SC fusion method presented in Section 3.2 will always select the UWB positions when both ZigBee and UWB technologies are covering the target at the same time, as UWB's accuracy is always better than ZigBee's.

**Figure 12.**
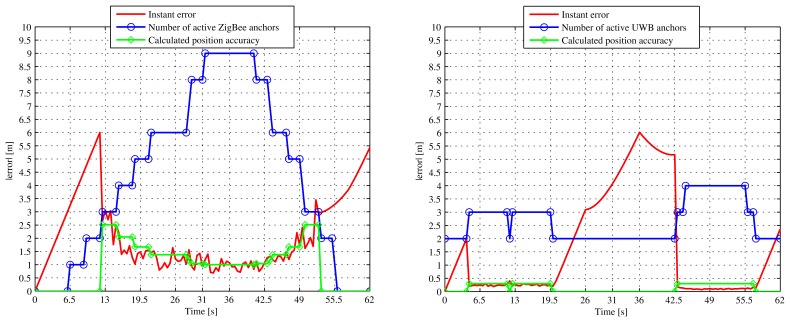
Instant error for the ZigBee and UWB networks when used separately.

Figure 12 shows the instant error of the simulated trajectory, after repeating it ten times to average the results (the ZigBee measurements are quite noisy). The instant error is calculated by the Euclidean distance (2-norm) between the real position of the target and the estimated position (calculated by the LS algorithm) at each time instant. Before each round starts, we always initialize the position estimation of the location algorithm at the (0,9) coordinate. In the subfigure on the left we show the case when the ZigBee node is used alone. In red we show the instant error, which is the absolute difference between the current estimated position and the real position of the ZigBee node. The first slope from 0 to about 6 meters of error is due to the lack of ZigBee coverage on the left-hand side of the room until the ZigBee anchors 4, 7 and 8 cover the mobile node (until it reaches the (6, 9) coordinate). Then, from this instant (*t* ≃ 12 s; until it loses coverage again (*t* ≃ 50 s), the ZigBee mobile node is detected from between 3 and 9 anchors at any one time. With a blue line in the figure, we represent the number of activated anchors with respect to time, and with a green line the calculated position accuracy defined in Equation (4;. Note that the *position.acc* is drawn as zero in the figure when there are no positions available. In fact, there are no positions stored in the server for such time intervals and, therefore, they have no associated *position.acc* value.

In the subfigure on the right side of Figure 12 we show the respective UWB case, when this technology is used alone. In this case the instant error falls to as low as 0.2 m when we have full coverage by the four UWB anchors. However, it lacks coverage during a long period of time, from *t* ≃ 19.5 s until *t* ≃ 42.5 s, when the error increases to as much as 6 m.

We can see that when both technologies are used independently we lack coverage during long periods of time, so that the error increases by up to several meters. By using the high-level fusion module of the proposed architecture, we can easily combine both technologies in a single multi-technology *target device* in a flexible manner to obtain fusion positions.

**Figure 13.**
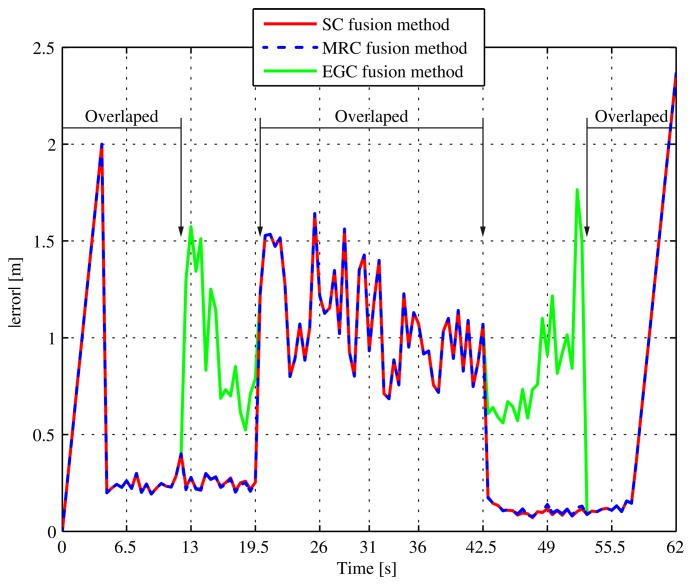
Instant error of the fusion positions when using the SC, MRC and EGC methods.

Figure 13 shows the instant error obtained when we ask the *Location Server* for fusion positions using the SC, MRC and EGC fusion methods. We see that the three fusion algorithms perform exactly the same when there is zero or only one technology available. This situation happens in the following time intervals: *t* ϵ (0,12) s (none or only UWB;, *t* ϵ (19.5, 42.5) s (only ZigBee; and *t* ϵ (50, 62) s (only UWB or none). In the rest of the time, when both technologies are available simultaneously, the SC method always select the most accurate technology available in terms of *position.acc* value, while MRC combines both technologies, assigning them weight depending again on the *position.acc* associated to the estimated position stored in the *Location Server.* Finally, EGC performs rather worse than the other two methods as it does not take into account the *position.acc* value for averaging the available positions. And, as soon as ZigBee positions become much noisier than UWB's, it produces worse fusion position estimations. As result, the proposed MRC and SC fusion methods perform very similarly in this simulation scenario because there is a huge difference in accuracy between ZigBee and UWB technologies. In a real scenario, possibly incorporating different RSS-based technologies, the MRC method would probably perform better, averaging a larger set of noisy data, but it is definitely dependent on the amount of anchors available and the indoor scenario characteristics.

The maximum combined instant error obtained with fusion data is less than 2.5 m throughout the whole simulation and is much better than using the WSN technologies separately.

Note that in all cases the fusion positions are pre-calculated in the *Location Server* before being sent back to the *Location Clients.* They are directly packed inside a JSON response message, and the clients only have to display them using an application with a GUI, on a map of the building.

Table 2 shows the minimum, mean and maximum size of the messages used during the simulation, in order to evaluate the bandwidth consumption for communicating with the location server. Both the new_measurement and new_position messages which insert data into the *Location Server* only receive a small OK acknowledge JSON message as a response, which occupies a fixed small amount of data. Other message responses such as the get_measurements and get_position can increase in size depending on the number of measurements or positions requested. In our simulations we always asked for positions in iterations every 0.5 s, for a window size of 0.499 s, so that the number of obtained positions was always zero or one for a specific technology (ZigBee or UWB), and for the fusion positions (zero or only one fusion position is given per call). This is the reason why the get_position responses do not grow much in this simulation. However, in the case of measurements, we ask for all the available measurements of the mobile nodes regarding all the anchors of each network, which can be from 1 to 9 measurements per response.

Note that during the simulations we have not used OpenID to authenticate the different clients with the *Location Server*. Therefore, the message size shown in Figure 2 shows exactly the size of the JSON messages going through the *Location Server*, not other network traffic involved in the authentication.

## 7. Conclusions

This paper introduces an RTLS architecture supporting multiple technologies. This architecture was designed to provide an easy means of including new hardware, new algorithms and any kind of LBS by considering client applications of the location estimations of targets of interest.

The architecture provides a complete API with several methods for interfacing with external modules. Hardware providers can set up a network in the platform based on their own technology in a transparent way, for example, to the algorithm developers. Algorithms can use raw data from one or more available technologies to provide a location estimation. The location estimations are available in real time (or off-line, with access to historical data) for location applications.

The advantage of the proposed architecture, as opposed to monolithic solutions, is that it allows a generalization and retargeting for new LBS and applications, reducing costs of further development. Other kind of purpose built systems can be expensive and hard to implement in practice.

To demonstrate the application of the architecture and its platform, the paper introduces two case studies based on real deployments. Moreover, the performance of the platform in a specific scenario has been demonstrated by the validation results.

## Figures and Tables

**Table 1.** Standardized types of measurement supported.

RSS	TimeStamp [ms]	AnchorID		MobileID	RSS [dBm]	Seq. Number
TOA	TimeStamp [ms]	AnchorID		MobileID	TOA [s]	Seq. Number
TDOA	TimeStamp [ms]	AnchorID_1_	AnchorID_2_	MobileID	TDOA_12_ [s]	Seq. Number
AOA	TimeStamp [ms]	AnchorID		MobileID	AOA [*α,β* rad]	Seq. Number
DISTANCE	TimeStamp [ms]	AnchorID		MobileID	Distance [m]	Seq. Number
SENSOR	TimeStamp [ms]	SensorID		Custom defined data*		

**Table 2.** Message size.

Message type	Call message size [bytes]			Response message size [bytes]		
	Minimum	Mean	Maximum	Minimum	Mean	Maximum

new measurement (ZigBee)	232	236	238	49	49	49
new measurement (UWB)	224	229	231	49	49	49

get measurement (ZigBee)	167	170	171	135	842	1639

get measurement (UWB)	164	167	168	447	566	785

new position (ZigBee)	221	225	228	55	55	55

new position (UWB)	216	219	221	55	55	55

get position (ZigBee)	219	222	223	159	282	350

get position (UWB)	216	219	220	156	242	338

get position (Fusion)	217	221	222	158	311	347
